# Evidence of internal structure of the transactional eHealth literacy among Vietnamese youth: An instrument validation study

**DOI:** 10.3389/fpubh.2023.1036877

**Published:** 2023-03-24

**Authors:** Thao Phuong Thi Nguyen, Anh Linh Do, Ha Ngoc Do, Thuc Minh Thi Vu, Robin van Kessel, Brian Li Han Wong, Laurent Boyer, Guillaume Fond, Pascal Auquier, Tham Thi Nguyen, Carl A. Latkin, Cyrus S. H. Ho, Roger C. M. Ho

**Affiliations:** ^1^Institute for Global Health Innovations, Duy Tan University, Da Nang, Vietnam; ^2^Faculty of Nursing, Duy Tan University, Da Nang, Vietnam; ^3^Institute of Health Economics and Technology, Hanoi, Vietnam; ^4^Youth Research Institute, Ho Chi Minh Communist Youth Union, Hanoi, Vietnam; ^5^Department of International Health, Care and Public Health Research Institute (CAPHRI), Maastricht University, Maastricht, Netherlands; ^6^Research Committee, Global Health Workforce Network (GHWN) Youth Hub, World Health Organization, Geneva, Switzerland; ^7^Medical Research Council Unit for Lifelong Health and Ageing at UCL, Department of Population Science and Experimental Medicine, UCL Institute of Cardiovascular Science, University College London, London, United Kingdom; ^8^EA 3279, CEReSS, Research Centre on Health Services and Quality of Life, Aix Marseille University, Marseille, France; ^9^Bloomberg School of Public Health, Johns Hopkins University, Baltimore, MD, United States; ^10^Department of Psychological Medicine, Yong Loo Lin School of Medicine, National University of Singapore, Singapore, Singapore; ^11^Institute for Health Innovation and Technology (iHealthtech), National University of Singapore, Singapore, Singapore

**Keywords:** eHealth, eHealth literacy, transactional model of eHealth literacy, digital health, validation

## Abstract

**Background:**

The progression into the Digital Age has brought an array of novel skill requirements. Unlike traditional literacy, there are currently few measures that can reliably measure eHealth literacy. The Transactional Model of eHealth Literacy and subsequent Transactional eHealth Literacy Instrument may provide a feasible option for measuring eHealth literacy.

**Objective:**

This instrument has yet to be validated, which is the aim of this study. In particular, this article was conducted to validate the TeHLI to see which components of the tool (how many and which components included) would be the best fit statistically and whether the tool applies to groups of different characteristics.

**Methods:**

We conducted an online cross-sectional study among 236 Vietnamese young people. A exploratory factor analysis was used to identify the best fit model of the Transactional eHealth Literacy Instrument. A confirmatory factor analysis tested measurement invariance at four levels: configural, metric, scalar, and strict invariance. Only metric invariance was partially invariant, while the rest tested fully invariant. Even with partial metric invariance, there is reason to assume that functional, communicative, critical, and translational eHealth literacy (the four levels according to the transactional model) are consistently measured when deploying the Transactional eHealth Literacy Instrument across groups.

**Results:**

The study findings substantiate that the most optimal composition of the TeHLI consists of four factors: functional, communicative, critical, and translational eHealth literacy, with RMSEA = 0.116; CFI = 0.907, and the highest internal consistency (Cronbach's α = 0.91, 0.92, 0.88, and 0.92 for each factor respectively). After using measurement invariance, that gender, education, marital status, age, location, and household economy do not influence the way participants to respond to the TeHLI to the point that would introduce measurement bias. In other word, using TeHLI across population groups should not produce error margins that substantially differ from each other.

**Conclusions:**

This study suggests the instrument can be used for comparisons across groups and has the potential to generate high-quality data usable for informing change agents as to whether a particular population is proficient enough to adopt novel eHealth innovations.

## 1. Introduction

eHealth literacy is a skill set that is becoming increasingly relevant as the Digital Age progresses (1, 2). When healthcare and public health services slowly moved to the digital sphere (3), the skills required to search for, identify, and access these services started changing incrementally (4). However, much like most of the world, these services transitioned to digital environments at the beginning of the COVID-19 pandemic (5). While innovations are typically taken up gradually by a population (6), society adapted acutely to the pandemic—resulting in digital and technological advances neither equally nor equitably permeating all layers of society across the globe and potentially widening existing inequalities (7–9). In fact, there are some indications that the migration to the digital environment was used as a short-term solution and that—when traditional health services reemerged—these were again preferred by those seeking healthcare (10).

eHealth literacy is a specific branch in the field of digital literacy (4). Given the transition toward digitalization globally, there have been attempts to define eHealth literacy. For instance, UNESCO frames digital literacy as a set of foundational skills needed to work in the digital world and is considered a catalyst for individuals to achieve health and social outcomes (11). Current training programs tend to focus heavily on technical skills required to use digital technologies, while cognitive and ethical considerations are disregarded entirely (12, 13). It takes ~5 years for the average individual to become sufficiently proficient in using digital technologies (12). However, this 5-year gap is not equal across all societal domains. For example, the medical sector has been slower to adapt to the digital world (14).

Recent work by Paige and colleagues created a tool with which to measure eHealth literacy (15). The Transactional eHealth Literacy Instrument (TeHLI) is a multi-dimensional instrument to measure increasing levels of eHealth literacy, based on the Transactional Model of eHealth Literacy (TMeHL) (16): functional (the ability to successfully read and write about health using technological devices), communicative (the ability to control, adapt, and collaborate communication about health with others in online social environments), critical (the ability to evaluate the relevance, trustworthiness, and risks of sharing and receiving health-related information on the Internet), and translational eHealth literacy (the ability to apply health-related information from the Internet in different contexts). However, given the novelty of this assessment tool, its application has only been focused on the general and middle age populations. However, as the driver population of eHealth utilization is the teenager and working population, more research is needed to test TeHLI on younger adults (15). This article aims to validate the TeHLI to see which components of the tool (how many components and which components included) would be the best fit statistically and whether the tool can be applied to groups of different characteristics (i.e., whether the different characteristics in terms of gender, education level, age group and other sociodemographic factors impact the effectiveness of the tool in any way) through exploratory and confirmatory factor analyses of survey data from Vietnamese young adults.

## 2. Materials and methods

### 2.1. Participants and study procedures

An online cross-sectional study was conducted among Vietnamese young people from April to June 2020 in Vietnam. The eligibility criteria for participating in this survey were: (1) ages between 16 and 35 years; (2) currently living in Vietnam; and (3) agreed to join this study by providing online informed consent.

In this study, the snowball sampling technique was used to recruit participants from all provinces of Vietnam. First, we developed a core group of participants with 20 people (including leaders of the Youth Union in different public institutions, companies, and organizations) and invited them to complete the survey. After completing the survey, these participants were requested to invite their peers in their respective networks to complete the online survey. At the end of the data collection period, 236 youths aged 16–35 living in Vietnam agreed to participate and completed the survey. This study was approved by the institutional review board of the Youth Research Institute, Vietnam.

### 2.2. Measurement and instrument

In this study, we built an online survey on the Survey Monkey platform. This approach is low cost, consumes little time, is user-friendly for youth, and is highly accessible to reach the samples nationwide. To complete this survey, each participant spent about 5–10 min. A structured questionnaire was used to collect information, consisting of two components: (1) sociodemographic characteristics, and (2) the TeHLI. The survey was first piloted on five youths to ensure the cross-cultural validity of translated instruments in Vietnamese. After that, the revised questionnaire was uploaded into the online survey portal. The data collection began once the online survey system was tested to assure accurate question contents and no technical issues.

#### 2.2.1. Sociodemographic and health status characteristics

Participants were asked to report their sociodemographic information, including age, sex (male/female), educational attainment (below high school and high school/college/tertiary and higher), marital status (single/other), and living areas (urban/town/rural or mountain area).

#### 2.2.2. Transactional eHealth literacy instrument

*The Transactional eHealth Literacy Instrument (TeHLI)* was developed to reflect the theoretical assumptions of the TMeHL by Paige et al. (15). This scale consisted 18 items to reflect four aspects of the TMeHL such as functional (4 items), communicative (5 items), critical (5 items), and translational (4 items). A 5-point Likert scale from 1 (strongly disagree) to 5 (strongly agree) was used for each item. The total score of all items in each domain was calculated, with 4–16 points for both functional and translational domains and 5–25 points for both communicative and critical domains. The higher score of each domain indicated a higher level of eHealth skills. In this study, the Cronbach's α of four domains of TeHLI were 0.91; 0.92; 0.88; 0.92, respectively. Furthermore, by reviewing previous studies, the characteristics, as well as other versions of TeHLI were present in Table 1.

**Table 1:** The characteristics of transactional ehealth literacy instrument (TeHLI).

**Authors/year of publication**	**Country**	**Aimed**	**No. of dimensions**	**Name of dimensions/number of items/dimensions**	**No. of items of instrument**	**Score range**
Paige et al. (15)	USA	Development and psychometric testing of a multi-dimensional Transactional eHealth Literacy instrument based on Transactional Model of eHealth Literacy (TMeHL)	4	(1) Functional (4 items) (2) Communicative (5 items) (3) Critical (5 items) (4) Translational (4 items)	18	5-point Likert-type scale: 1: Strongly disagree 2: Disagree 3: Undecided 4: Agree 5: Strongly agree
Taylor et al. (17)	USA	(1) Validate the TeHLI within a sample of caregivers of patients living with a blood cancer	4	(1) Functional (4 items) (2) Communicative (5 items) (3) Critical (5 items) (4) Translational (4 items)	18	5-point Likert-type scale: 1: Strongly disagree 2: Disagree 3: Undecided 4: Agree 5: Strongly agree
		(2) Validate the TeHLI after adding a 5th dimension within a sample of caregivers of patients living with a blood cancer	5	(1) Functional (4 items) (2) Communicative (5 items) (3) Critical (5 items) (4) Translational (4 items) (5) Clinical (5 items)	23	5-point Likert-type scale: 1: Strongly disagree 2: Disagree 3: Undecided 4: Agree 5: Strongly agree

### 2.3. Statistical analysis

Statistical analysis was performed using STATA version 16 and R. Standard descriptive statistical analysis was conducted with mean and standard deviation (SD) for quantitative variables and frequency and percentage for qualitative variables. The value of Skewness and Kurtosis coefficients were reported. Floor and ceiling effects were identified if the percentage of participants answering the lowest or highest response option was above 15% (18). A *p*-value (*p*) < 0.05 was considered statistically significant. The Exploratory Factor Analysis (EFA) was performed to indicate the items belonging to the four models of the TMeHL: Model 1 (18 items divided into one factor); Model 2 (18 items into two factors); Model 3 (18 items into three factors); Model 3 (18 items into four factors). For the estimation of the number of questions of each component and properties of each item in each model, an orthogonal varimax rotation with Kaiser normalization was utilized. Furthermore, after using EFA, to determine the factor structure, we conducted a Confirmatory Factor Analysis (CFA) to test the original model (4 factors), then the new models of TeHLI. Multiple model fit indicators with respective cutoffs assessed the model fit of observed data (with Satorra-Bentler correction for non-normality data) (19), including:

- Relative Chi-square (χ^2^/*df*): a value ≤3.0 for good fit;
- Root Mean Square Error of Approximation (RMSEA): a value of ≤0.08 for good fit;
- Comparative Fit Index (CFI): a value of ≥0.9 for acceptable fit;
- Standardized Root Mean Square Residual (SRMR): a value of ≤0.08 for good fit.

#### 2.3.1. Measurement invariance

Measurement invariance of 4-factor model of the TeHLI regarding gender, age group, education, marital status, location, and household economy was tested by using four models as recommended. four models with different levels of measurement invariance: (1) configural invariance; (2) metric invariance; (3) strong (scalar) invariance; and (4) strict invariance.

Firstly, we evaluated *configural* invariance to assess what model is least stringent. This step aimed to test whether the constructs have the same pattern of free and fixed loadings across groups. Secondly, when configural invariance was supported, we evaluated *metric* invariance (also called pattern or weak invariance) or equivalence of the item loadings on the factors. Metric invariance was established by constraining factor loadings to be equivalent across groups. Thirdly, we investigated *scalar* invariance by constraining equal intercepts and factor loadings across groups. This step assures that participants in different groups, on average, rate items similarly. Finally, we analyzed *strict* invariance, which is supported when equal error variances are constrained in addition to equal intercepts and factor loadings. Testing residual error establishes that the same amount of error, or variance not accounted by the factor, is consistent for each item across groups. Measurement invariance is tested by some goodness of fit indexes, such as ΔCFI, ΔRMSEA, Δχ^2^. When we used Δχ^2^ as a sole measure of exact fit, the results showed that it was associated with lower levels of scalar invariance. By contrast, using the ΔCFI (with or without other criteria like ΔRMSEA) was associated with higher levels metrics, scalar, and strict model. Therefore, in this study, change in the CFI (ΔCFI) was used as the primary criterion for comparing models, and ΔCFI < 0.01 between successively more restricted models provides evidence for measurement invariance (20–22).

## 3. Results

Table 2 shows the sociodemographic of the 236 participants in this study. The mean age of respondents was 21.3 ± 4.8 years, with the most common age group about above 24 years old (78.8%). The majority of those were female (71.2%) and 56.8% of participants had the education tertiary and upper.

**Table 2:** Demographic characteristics of participants.

**Characteristics**	***n***	**%**
**Total**	236	100.0
**Gender**		
Male	68	28.8
Female	168	71.2
**Age group**		
Below 24-year	186	78.8
Above 24-year	50	21.2
**Education**		
Below and high school	59	25.0
College	43	18.2
Tertiary and higher	134	56.8
**Location**		
Urban	110	46.6
Suburban	41	17.4
Rural/mountain area	85	36.0
**Household economy**		
Poor	136	57.6
Medium and above	100	42.4
	**Mean**	**SD**
Age	21.3	4.8

Table 3 presents the results of the descriptive analysis for 18 items of the TeHLI. All the 18 items had a range of scores from 1 to 5. Out of 18 items, the maximum and mimimum scores was recored at 3.3 (0.9), and 3.3 (0.9), respectively. Skewness and Kurtosis coefficients were reported with ranging from −1.0 to −0.2 and 2.8 to 4.3, respectively. Table 3 also presented the reliability of the modified TeHLI. Correlation coefficients with other items in respective factors ranged between 0.7 and 0.8. Cronbach's alpha value of factor 1 “Functional,” factor 2 “Communicative,” factor 3 “Critical,” and factor 4 “Translational” were good at 0.82, 0.92, 0.92, and 0.90, respectively.

**Table 3:** Basic descriptions and reliability of transactional eHealth literacy.

**Items**	**Responses (%)**					**Mean (SD)**	**Skewness**	**Kurtosis**	**Floor (%)**	**Ceiling (%)**	**Item-total correlation**	**Cronbach's alpha if item deleted**
	**1**	**2**	**3**	**4**	**5**							
**Factor 1: Functional**												
Q1. Summarize basic health information	4.2	12.3	36.0	39.0	8.5	3.4 (0.9)	−0.5	3.0	4.2	8.5	0.7	0.96
Q2. Know how to access basic health information	3.0	6.8	22.5	58.1	9.8	3.6 (0.9)	−1.0	4.3	3.0	9.8	0.7	0.96
Q3. Create messages that describe health	3.0	9.3	34.8	45.8	7.2	3.4 (0.9)	−0.6	3.4	3.0	7.2	0.7	0.96
Q4. Tell someone how to find basic health information	3.0	8.5	29.7	49.2	9.8	3.5 (0.9)	−0.7	3.5	3.0	9.8	0.8	0.96
**Factor 2: Communicative**												
Q5. Information goals and help others have it	3.4	9.8	33.1	44.1	9.8	3.5 (0.9)	−0.6	3.2	3.4	9.8	0.8	0.96
Q6. Talk about health topics with users	3.4	11.9	41.5	35.2	8.1	3.3 (0.9)	−0.3	3.1	3.4	8.1	0.8	0.96
Q7. Identify the emotional tone of a health conversation	3.4	11.4	43.2	35.6	6.4	3.3 (0.9)	−0.4	3.2	3.4	6.4	0.8	0.96
Q8. Contribute to health conversations	2.5	14.0	43.2	32.6	7.6	3.3 (0.9)	−0.2	2.9	2.5	7.6	0.8	0.96
Q9. Connections with others to share information	2.5	8.9	40.7	40.7	7.2	3.4 (0.8)	−0.4	3.4	2.5	7.2	0.8	0.96
**Factor 3: Critical**												
Q10. Identify a credible source of health information	2.5	15.3	39.8	35.2	7.2	3.3 (0.9)	−0.2	2.8	2.5	7.2	0.7	0.96
Q11. Identify health information is fake	3.8	10.6	41.5	36.9	7.2	3.3 (0.9)	−0.4	3.2	3.8	7.2	0.8	0.96
Q12. Identify website is safe for sharing personal health	4.2	10.6	39.8	38.6	6.8	3.3 (0.9)	−0.5	3.2	4.2	6.8	0.7	0.96
Q13. Identify information is relevant to health needs	3.0	6.4	38.6	45.3	6.8	3.5 (0.8)	−0.6	3.8	3.0	6.8	0.8	0.96
Q14. Know how to evaluate the credibility of others	3.4	11.4	39.8	39.4	5.9	3.3 (0.9)	−0.5	3.2	3.4	5.9	0.8	0.96
**Factor 4: Translational**												
Q15. Learn to manage health in a positive way	3.4	8.9	36.0	44.1	7.6	3.4 (0.9)	−0.6	3.4	3.4	7.6	0.8	0.96
Q16. Use the Internet as a tool to improve health	3.4	13.6	36.0	39.4	7.6	3.3 (0.9)	−0.4	2.9	3.4	7.6	0.8	0.96
Q17. Use the information to make a decision	3.8	13.1	40.7	35.2	7.2	3.3 (0.9)	−0.3	3.0	3.8	7.2	0.8	0.96
Q18. Learn about topics had relevant to me	3.8	8.1	30.9	46.6	10.6	3.5 (0.9)	−0.7	3.5	3.8	10.6	0.7	0.96
**Factor score**												
**Factor 1: Functional**						3.5 (0.8)	−0.8	4.1				0.88
**Factor 2: Communicative**						3.4 (0.8)	−0.3	3.7				0.92
**Factor 3: Critical**						3.4 (0.8)	−0.4	3.4				0.92
**Factor 4: Translational**						3.4 (0.8)	−0.5	3.4				0.90
**All**						3.4 (0.7)	−0.4	4.0				0.96

Table 4 further shows the results of Confirmatory Factor Analysis on four different models. The model with 1 factor of the TeHLI showed an RMSEA and CFI score of 0.157 and 0.806. In contrast, the model with four factors showed an RMSEA and CFI score of 0.116 and 0.907, respectively. As such, this model was used to conduct measurement invariance.

**Table 4:** Exploratory factor analysis and confirmatory factor analysis models and fit indices evaluating factor structure of the transactional eHealth literacy scale.

	**Model 1**	**Model 2**	**Model 3**	**Model 4**
Q1. Summaries basic health information	eHealth literacy	Functional/communicative	Functional	Functional
Q2. Know how to access basic health information	eHealth literacy	Functional/communicative	Functional	Functional
Q3. Create messages that describe health	eHealth literacy	Functional/communicative	Functional	Functional
Q4. Tell someone how to find basic health information	eHealth literacy	Functional/communicative	Functional	Functional
Q5. Information goals and help others have it	eHealth literacy	Functional/communicative	Communicative	Communicative
Q6. Talk about health topics with users	eHealth literacy	Functional/communicative	Communicative	Communicative
Q7. Identify the emotional tone of a health-related conversation	eHealth literacy	Functional/communicative	Communicative	Communicative
Q8. Contribute to health conversations	eHealth literacy	Functional/communicative	Communicative	Communicative
Q9. Connections with others to share information	eHealth literacy	Functional/communicative	Communicative	Communicative
Q10. Identify a credible source of health information	eHealth literacy	Critical/translational	Critical/translational	Critical
Q11. Identify health information is fake	eHealth literacy	Critical/translational	Critical/translational	Critical
Q12. Identify a website that is safe for sharing personal health	eHealth literacy	Critical/translational	Critical/translational	Critical
Q13. Identify information is relevant to health needs	eHealth literacy	Critical/translational	Critical/translational	Critical
Q14. Know how to evaluate the credibility of others	eHealth literacy	Critical/translational	Critical/translational	Critical
Q15. Learn to manage health in a positive way	eHealth literacy	Critical/translational	Critical/translational	Translational
Q16. Use the Internet as a tool to improve health	eHealth literacy	Critical/translational	Critical/translational	Translational
Q17. Use the information to make a decision	eHealth literacy	Critical/translational	Critical/translational	Translational
Q18. Learn about topics had relevant to me	eHealth literacy	Critical/translational	Critical/translational	Translational
Chi-square	912.388	765.282	674.437	488.49
Degrees of freedom	135	134	132	117
*p*-value	<0.01	<0.01	<0.01	<0.01
RMSEA (90% CI)	0.157 (0.147; 0.166)	0.142 (0.132; 0.151)	0.132 (0.122; 0.142)	0.116 (0.106; 0.127)
TLI	0.780	0.82	0.843	0.897
CFI	0.806	0.842	0.865	0.907

RMSEA, root mean square error of approximation; TLI, Tucker-Lewis index; CFI, comparative fit index.

**Figure 1:**
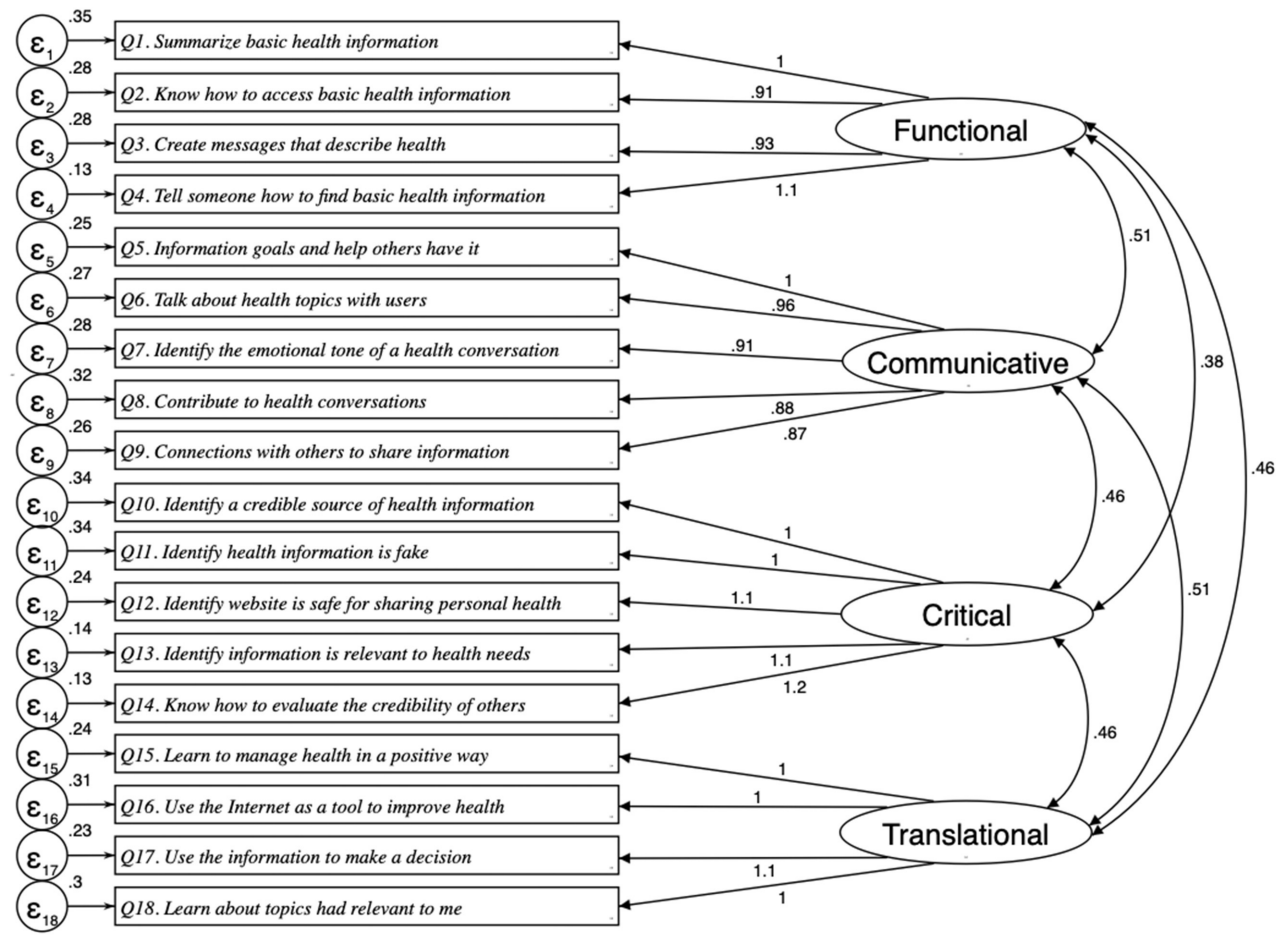
CFA models through structural equation modeling for the transactional eHealth literacy instrument (RMSEA = 0.116; CFI: 0.907; SRMR: 0.047).

The results obtained from the Confirmatory Factor Analysis for the TeHLI are estimated in Figure 1. The standardized coefficients range from 0.87 to 1.1. The highest coefficient was found in 4 items (Q4, Q11, Q13, and Q17), while the lowest was Q8.

The values of ΔCFI were <0.01 when all models, with progressive restrictions, are compared across age groups, education, household economic (Table 5). These groups reported strict invariance. Besides, the configural model of gender, marital status, and location had fit statistics of RMSEA < 0.08, CFI > 0.9. However, in the metric model, the value of ΔCFI was higher than 0.01 at all three variables (gender, marital status, and location). Metric invariance was therefore not supported by the data.

**Table 5:** Measurement invariance in the transactional eHealth literacy questionnaire for the different groups.

**Group**	**Invariance**	***x*^2^ (df)**	***x*^2^/*df***	**CFI**	**RMSEA (90%CI)**	**Δ *x*^2^(Δ df)**	**ΔCFI**	**Δ RMSEA**
Gender	Configural	288.467 (258)	1.118	0.966	0.032 (0.000; 0.050)	–	–	–
	Metric	289.257 (272)	1.063	0.981	0.023 (0.000; 0.044)	0.790 (14)	0.015	0.009
	Strong	307.891 (286)	1.077	0.976	0.026 (0.000; 0.045)	18.634 (14)	0.005	0.003
	Strict	326.862 (304)	1.075	0.975	0.025 (0.000; 0.045)	18.971 (18)	0.001	0.001
Education	Configural	406.792 (387)	1.051	0.978	0.026 (0.000; 0.049)	–	–	–
	Metric	436.604 (415)	1.052	0.976	0.026 (0.000; 0.048)	29.812 (28)	0.002	0.000
	Strong	466.686 (443)	1.053	0.974	0.026 (0.000; 0.048)	30.082 (28)	0.002	0.000
	Strict	501.197 (479)	1.046	0.975	0.024 (0.000; 0.046)	34.511 (36)	0.001	0.002
Marital status	Configural	286.936 (258)	1.112	0.964	0.031 (0.000; 0.050)	–	–	–
	Metric	288.11 (272)	1.059	0.98	0.022 (0.000; 0.044)	1.174 (14)	0.016	0.008
	Strong	300.773 (286)	1.052	0.982	0.021 (0.000; 0.042)	12.663 (14)	0.002	0.001
	Strict	316.362 (304)	1.041	0.985	0.019 (0.000; 0.041)	15.589 (18)	0.003	0.002
Age group	Configural	278.739 (258)	1.080	0.977	0.026 (0.000; 0.046)	–	–	–
	Metric	282.998 (272)	1.040	0.988	0.019 (0.000; 0.042)	4.259 (14)	0.010	0.008
	Strong	297.374 (286)	1.040	0.987	0.018 (0.000; 0.041)	14.376 (14)	0.001	0.001
	Strict	314.93 (304)	1.036	0.988	0.018 (0.000; 0.040)	17.556 (18)	0.001	0.000
Location	Configural	415.767 (387)	1.074	0.967	0.031 (0.000; 0.052)	–	–	–
	Metric	419.695 (415)	1.011	0.995	0.012 (0.000; 0.042)	3.928 (28)	0.028	0.019
	Strong	450.521 (443)	1.017	0.991	0.015 (0.000; 0.042)	30.826 (28)	0.003	0.003
	Strict	484.828 (479)	1.012	0.993	0.013 (0.000; 0.041)	34.307 (36)	0.002	0.002
Household economy	Configural	285.653 (258)	1.107	0.969	0.030 (0.000; 0.049)	–	–	–
	Metric	303.942 (272)	1.117	0.964	0.032 (0.000; 0.050)	18.289 (14)	0.005	0.002
	Strong	317.675 (286)	1.111	0.964	0.031 (0.000; 0.049)	13.733 (14)	0.000	0.001
	Strict	334.803 (304)	1.101	0.965	0.029 (0.000; 0.047)	17.128 (18)	0.001	0.002

## 4. Discussion

This article aimed to validate the TeHLI to see which components of the tool (how many and which components included) would be the best fit statistically and whether the tool applies to groups of different characteristics. Overall, we found that a TeHLI divided into four factors (functional, communicative, critical, and translational) had the best statistical fit (RMSEA = 0.116; CFI = 0.907) and the highest level of internal consistency (Cronbach's α = 0.96). The individual items in the TeHLI have medium-to-high levels of correlation with other items in respective factors (*r* > 0.6). Upon further analysis, we found partial invariance of the TeHLI—only lacking support for metric invariance.

The study findings substantiate that the most optimal composition of the TeHLI consists of four factors: functional, communicative, critical, and translational eHealth literacy. In this model, Q1–Q4 measure functional eHealth literacy, Q5–Q9 measure communicative eHealth literacy, Q10–Q14 measure critical eHealth literacy, and Q15–Q18 measure transactional eHealth literacy. This tool composition is in line with the original dimensions of the TMeHL (16). The initial measurements of high internal consistency of the four domains by Paige and colleagues (Cronbach's α = 0.91, 0.92, 0.88, and 0.92 respectively) was also supported by our findings (Cronbach's α = 0.88, 0.92, 0.92, and 0.90 respectively) (15).

In terms of measurement invariance, the support for configural invariance indicates that the same construct is measured in different population groups. In the context of this study, functional, communicative, critical, and translational eHealth literacy are consistently measured when the TeHLI is deployed in different population groups. The support for scalar invariance indicates the ability to justify the comparison of means across population groups. It indicates that gender, education, marital status, age, location, and household economy do not influence the way participants to respond to the TeHLI to the point that would introduce measurement bias. The support for strict invariance indicates that the residual errors are equivalent across participants. In other words, using the TeHLI across population groups should not produce error margins that substantially differ from each other. However, metric invariance was only found in two groups (education [ΔCFI = 0.002] and household economy [ΔCFI = 0.005]; age group being precisely on the threshold [ΔCFI = 0.010]) and, therefore, only partial metric invariance is supported by this data (23), which suggests that the constructs measured have different meanings across some of the participant groups.

Finding partial support for metric invariance is reported to have minimal effects on the mean differences of a latent factor (24). Since two factor loadings reported metric invariance, there are grounds to assume the TeHLI can be used for comparisons across groups (25, 26). Simultaneously, there is also the question of whether partial invariance is sufficient as significant bias can still be introduced into study findings if the partial invariance is ignored (27, 28). As such, deploying a partially (including invariant items only) and a fully invariant model (including invariant and non-invariant items) and compare the results of interest arises as a viable solution to use the TeHLI across different population groups (29). The risk of this solution is that—if the results differ substantially—there is no clear path forward.

Some limitations apply to this study. The survey findings had a mild-to-moderate negative skewness. However, as extrapolation to the population is impossible given the lack of participants, this limitation does not significantly affect the study findings. The survey was also distributed only among the Vietnamese population. Therefore, the influence of culture could not be determined and needs to be examined in a future study that includes multiple countries or regions. Lastly, the current study sample did not reflect a balanced distribution of some socio-demographic characteristics and could lead to limit representativeness with respect to the Vietnamese youth population. Hence, further studies are required to explore the effect of the data imbalance on measurement invariances of the scales.

## 5. Conclusions

Ultimately, the TeHLI can be considered a valuable tool to measure different competency levels of eHealth literacy. As such, the TeHLI has the potential (if deployed correctly) to generate high-quality data that can be used to inform governments and change agents whether the general or a target population is capable of understanding, appraising, distinguishing, and acting appropriately on high- and low-quality health information on the Internet. As a result, it can make vital contributions in informing and guiding policy and practice sustainably into the Digital Age.

## Data availability statement

The raw data supporting the conclusions of this article will be made available by the authors, without undue reservation.

## Ethics statement

The protocol of this study was approved by the Institutional Review Board of Youth Research Institute, Vietnam (No 404QÐ*/TWÐ*TN-VCNTN) on 08/01/2020. The patients/participants provided their written informed consent to participate in this study.

## Author contributions

TPTN: conceptualization, data collection, project management, and writing—review and editing. AD and HD: conceptualization, data collection, and project management. TV and RK: formal analysis, validation, writing—original draft, and writing—review and editing. BLHW, LB, GF, and PA: formal analysis, writing—original draft, and writing—review and editing. TTN: formal analysis, data collection, data analysis, and writing—original draft. CL and CH: conceptualization and writing—review and editing. RH: conceptualization, validation, and writing—review and editing. All authors contributed to the article and approved the submitted version.
